# Neurodevelopmental outcomes of infants born to mothers with SARS-CoV-2 infections during pregnancy: a national prospective study in Kuwait

**DOI:** 10.1186/s12887-022-03359-2

**Published:** 2022-05-30

**Authors:** Mariam Ayed, Alia Embaireeg, Mais Kartam, Kiran More, Mafaza Alqallaf, Abdullah AlNafisi, Zainab Alsaffar, Zainab Bahzad, Yasmeen Buhamad, Haneen Alsayegh, Wadha Al-Fouzan, Hessa Alkandari

**Affiliations:** 1 Neonatal Department, Farwaniya Hospital, 81400 Kuwait City, Kuwait; 2 Paediatric Department, Farwaniya Hospital, 81400 Kuwait City, Kuwait; 3 Division of Neonatology, Sidra Medicine, Doha, Qatar; 4 Paediatric Department, Adan Hospital, Hadiya, Kuwait; 5 Paediatric Department, Sabah Hospital, Kuwait City, Kuwait; 6 Paediatric Department, Amiri Hospital, Kuwait City, Kuwait; 7 Department of Microbiology, Faculty of Medicine, Kuwait University, Jabriya, Kuwait; 8 Population Health Department, Dasman Diabetes Institute, Kuwait City, Kuwait

**Keywords:** Coronavirus 2019 (SARS-CoV-2), Pregnancy, Neurodevelopment of infants, Perinatal transmission

## Abstract

**Background:**

An increasing proportion of women are infected with severe acute respiratory syndrome coronavirus 2 (SARS-CoV-2) during pregnancy. Intrauterine viral infections induce an increase in the levels of proinflammatory cytokines, which inhibit the proliferation of neuronal precursor cells and stimulate oligodendrocyte cell death, leading to abnormal neurodevelopment. Whether a maternal cytokine storm can affect neonatal brain development is unclear. The objective of the present study was to assess neurodevelopmental outcomes in neonates born to mothers with SARS-CoV-2 infections during pregnancy.

**Methods:**

In this prospective cohort study, the neurodevelopmental status of infants (*N* = 298) born to women with SARS-CoV-2 infections during pregnancy was assessed at 10–12 months post-discharge using the Ages and Stages Questionnaire, 3rd edition (ASQ-3). The ASQ-3 scores were classified into developmental delays (cutoff scores ≤ 2 standard deviations (SDs) below the population mean) and no delays (scores > 2 SDs above the population mean).

**Results:**

The majority (90%) of the infants born to mothers with SARS-CoV-2 infections during pregnancy had favorable outcomes and only 10% showed developmental delays. Two of the 298 infants tested positive for SARS-CoV-2, and both had normal ASQ-3 scores. The majority of the pregnant women had SARS-CoV-2 infections during their third trimester. The risk of developmental delays among infants was higher in those whose mothers had SARS-CoV-2 infections during the first (*P* = 0.039) and second trimesters (*P* = 0.001) than in those whose mothers had SARS-CoV-2 infections during the third trimester.

**Conclusion:**

The neurodevelopmental outcomes of infants born to mothers with SARS-CoV-2 infections seem favorable. However, more studies with larger sample sizes and longer follow-up periods are required.

## Background

Severe acute respiratory syndrome coronavirus 2 (SARS-CoV-2), which causes coronavirus disease 2019 (COVID-19), has been spreading rapidly worldwide, increasingly affecting pregnant females. Pregnancy-associated physiological changes, as well as altered cell-mediated immunity, enhance the susceptibility of pregnant women to infections by intracellular organisms such as viruses. Based on a living systematic review and meta-analysis, 10% (95% confidence interval (CI): 7–12%; 73 studies involving 67,271 women) of the pregnant and recently pregnant women attending or admitted to the hospital for any reason were diagnosed as having suspected or confirmed SARS-CoV-2 infections [1]. Hence, a growing proportion of pregnant women are infected with SARS-CoV-2 during various trimesters of pregnancy, with variable effects on the fetuses [1, 2].

Furthermore, the underdeveloped innate and adaptive immune systems of fetuses and neonates also make them more susceptible to infections [3, 4]. Maternal–fetal transmission of SARS-CoV-2 has not been widely established to date, and the findings vary. A meta-analysis revealed that vertical transmission of SARS-CoV-2 is possible and showed that only 3.2% (27 of 936; 95% CI: 2.2–4.3) of the neonates from mothers infected with SARS-CoV-2 had a positive result based on viral RNA testing of nasopharyngeal swabs [5]. SARS-CoV-2 viral RNA testing was positive in neonatal cord blood (2.9% of the samples), placental swabs (7.7%), and fecal or rectal swabs, whereas it was negative in urine and amniotic fluid samples. In contrast, a more recent systematic review article, which covered the database up to September 2020, reported that newborn rates of the infection vary between 0% and 11.5% [6]. Nevertheless, due to the availability of only a few patients and studies, the exact rates of vertical transmission and fetal or neonatal morbidity and mortality cannot be ascertained.

Although SARS-CoV-2 primarily causes respiratory distress, it is also known to have extrapulmonary manifestations, including hematological, cardiovascular, endocrinological, and neurological complications, in the adult population [7–9]. Of these, neurological manifestations in pregnant women and their probable effects on fetuses and neonates are of primary concern. SARS-CoV-2 may gain entry into the central nervous system through the nasal mucosa, lamina cribrosa, and olfactory bulb or through retrograde axonal transport [8]. SARS-CoV-2 also exhibits neurovirulence, triggering proinflammatory and prothrombotic cascades in the wake of cytokine storms, affecting brain vasculature, as well as the blood–brain barrier, mainly in the setting of the toxic-metabolic sequelae of multiorgan dysfunction that is frequently observed in SARS-CoV-2-positive patients [10, 11]. Proinflammatory cytokines inhibit the proliferation of neuronal precursor cells, activate astrogliosis, and stimulate oligodendrocyte cell death, leading to abnormal neurodevelopment [3, 4]. Moreover, the elevated levels of maternal inflammatory cytokines (IL-1, IL-6, IL-8, and TNF-α), as a consequence of infection during pregnancy, can disturb several characteristics of fetal brain development [12]. Notably, these pathological changes, which include deteriorated neuronal functions and atypical behavioral changes, can subsequently be seen in postnatal life and may increase the risk of schizophrenia, autism-related behavior, and mental disorders, including decreased sensorimotor gating, deficits in working memory, deficits in cognitive flexibility, and increased anxiety [12–16].

Because of the plausible effects on neonates born to mothers with SARS-CoV-2 infections during pregnancy, this study assessed the developmental attainment of neonates born to a cohort of mothers with SARS-CoV-2 infections during pregnancy in Kuwait at 10–12 months using the Ages and Stages Questionnaire, 3rd edition (ASQ-3).

## Methods

### Participants

In this prospective cohort study, infants born between April 1 and December 30, 2020, to mothers with SARS-CoV-2 infections during various trimesters of pregnancy were evaluated. Pregnant women who tested positive by reverse transcription-polymerase chain reaction (RT–PCR) for SARS-CoV-2 (Cobas 6800 Systems, Roche, Switzerland)/(TaqPath, Thermo-Fisher Scientific, USA) were identified from the Kuwait National COVID-19 registry. During the study period, Kuwait’s national COVID-19 policy implemented universal SARS-CoV-2 screening for all delivering mothers by nasopharyngeal PCR. Moreover, neonates born to SARS-CoV-2-positive mothers underwent SARS-CoV-2 nasopharyngeal swab testing at 48–72 h of age.

Mothers with equivocal RT–PCR test results and those with missing data were not included in the study. Verbal informed consent was obtained from all the parents who participated in the study. Ethics approval was granted by the Ethics Committee of the Ministry of Health of Kuwait (2021–1638), Government of Kuwait.

Demographic details were collected for all infants enrolled in the follow-up study. Maternal clinical and neonatal data from acute hospital admissions for the included participants were collected by retrospective chart review and have been previously published [17]. For maternal data, we collected information on maternal age, parity, educational level, gestational age at diagnosis of SARS-CoV-2 infection and presenting symptoms of COVID-19. We defined gestational age as the number of weeks calculated according to the last normal menstrual period or the expected date of delivery according to an early ultrasound scan. We classified gestational age at diagnosis of SARS-CoV-2 infection as the first trimester (less than 13 weeks’ gestation), second trimester (13–26 weeks’ gestation) and third trimester (more than 26 weeks’ gestation). For neonatal data, we collected information on gestational age at delivery, mode of delivery, birth weight, neonatal intensive care hospitalization and the result of SARS-CoV-2 real-time PCR on nasopharyngeal swabs at 48–72 h of age.

### Instrument

The ASQ-3 (http://agesandstages.com/) is a widely used screening tool for assessing development in children aged 1–66 months at a low cost, with cutoff scores identifying developmental delays. The screening tool has strong validity, 2-week test–retest reliability, interobserver reliability, and internal consistency [18]. The ASQ-3 has five domains: Communication, gross motor skills, fine motor skills, problem-solving capacity, and personal-social development.

In the current study, we used the ASQ-3 to assess neurodevelopmental outcomes at 10–12 months post-discharge. The infants’ parents completed the ASQ-3, which has a series of 21 developmental screening questions. Three study investigators (MAL, MK, and AE) scored the questionnaire results using the ASQ-3 age-specific scoring sheet. In each domain, six items were scored as shown by the infant, sometimes shown, or not yet shown by the infant, with 10, 5, and 0 points, respectively. If an item was not completed, a score of 0 was assigned based on the lowest possible result. Responses were summed to determine a score of 0 to 60 for each domain, with an overall maximum ASQ-3 score of 300 points. The ASQ-3 results were categorized according to the questionnaire-defined subscale cutoff scores: 1) developmental delays, with an ASQ-3 cutoff score of ≤ 2 standard deviations (SDs) below the population mean for 1 or more domains and 2) no delays, with a cutoff score > 2 SDs above the population mean [18].

The questionnaire was sent in two languages: The Arabic version for Arabic-speaking parents and the English version for non-Arabic-speaking parents. The questionnaire was completed by the parents and reviewed in a telephone interview with an investigator. The involved investigator was trained to administer the ASQ-3 and to help parents complete the questionnaire. The parents were encouraged to provide their responses with their interpretation without prompting. Parental informed consent was obtained from each subject prior to participation in the study.

### Statistical analysis

All data were entered into a Microsoft Excel database. Categorical variables are summarized as counts (n) and percentages (%), and continuous variables are summarized as the median and interquartile range (IQR). Statistical comparisons between the groups were performed using the chi-squared test for categorical variables and the Wilcoxon rank-sum test for continuous variables.

We performed a multivariate logistic regression analysis for each potential predictor of developmental delays. In the regression model, only variables with a *P* value of < 0.1 in the univariate analysis (maternal age, trimester at SARS-CoV-2 infection and gestational age at delivery) and potential confounders such as birth weight, sex, parental education and type of feeding for the first 6 months of age were included. The results of the regression analysis are presented as an adjusted odds ratio (aOR) with a 95% CI. Statistical significance was set at *P* < 0.05. Statistical analysis was performed with STATA 14 software (Stata Corporation, College Station, TX).

### Results

**Fig. 1:**
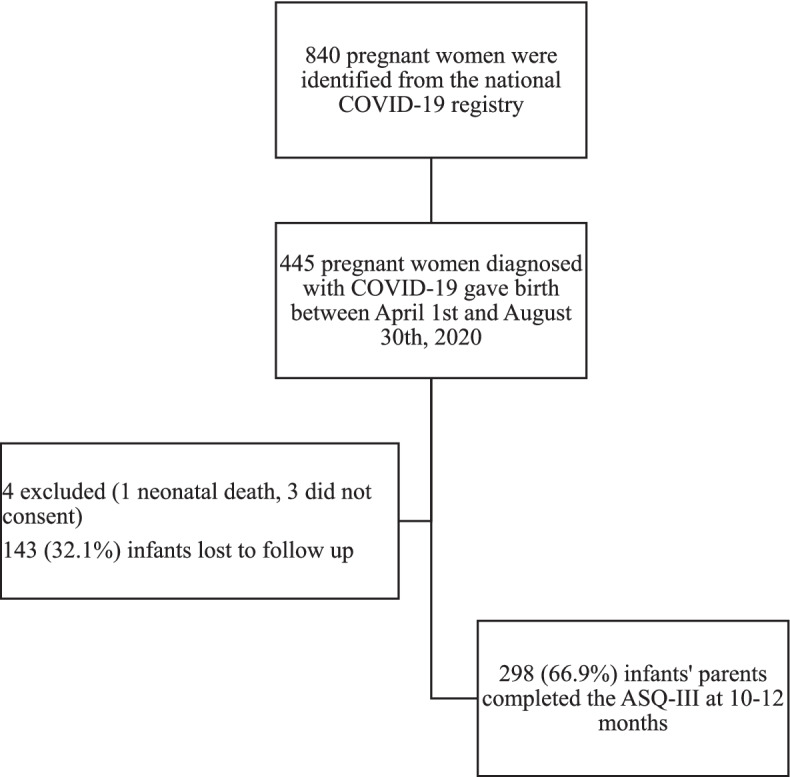
Flowchart of the study recruitment and follow-up. ASQ-3: Ages and Stages Questionnaire, 3rd edition

Eight hundred forty pregnant women were identified from the Kuwait National COVID-19 registry between April 1 and December 30, 2020. Four hundred forty-five women gave birth during the study period (Fig. 1). One infant died on the first day of life due to severe birth asphyxia; 298 (67%) had complete follow-up at 10–12 months corrected age, and 146 (32.8%) infants were lost to follow-up. The main reason for the loss to follow-up was the inability to contact the parents due to either invalid contact information or the parents having left the country. To determine whether the infants lost to follow-up were representative of the complete study and to avoid attrition bias, we compared the baseline features of the infants with follow-up data with the baseline features of the infants who were lost to follow-up. We did not find any statistically significant differences between these two groups.

The maternal and neonatal characteristics of the cohort are shown in Table 1.

**Table 1:** Maternal and neonatal demographic and clinical characteristics

Variable			All (*N* = 298)		
**Maternal characteristics, N (%) or median (IQR)**					
Age (years)			31 (27–35)		
Parity			3 (2–4)		
Gestational diabetes			51 (17.1%)		
Pregnancy-induced hypertension			26 (8.7%)		
**Maternal nationality**					
Kuwaiti		112 (37.6%)	Non-Kuwaiti		186 (62.4%)
**Maternal educational level**					
Educated		297 (99%)	Non-educated		3 (1%)
Elementary school		8 (2.7%)	Middle school		49 (16.4%)
High school		38 (12.7%)	Diploma holder		47 (15.8%)
Bachelor’s		147 (49.3%)	Master’s and PhD		6 (2%)
**Paternal educational level**					
Educated		297 (99.3%)	Non-educated		2 (0.7%)
Elementary school		6 (2%)	Middle school		41 (13.8%)
High school		66 (22.2%)	Diploma holder		57 (19.2%)
Bachelor’s		119 (40.1%)	Master’s and PhD		6 (2%)
**Gestational age at SARS-CoV-2 infection**					
*** 1***^***st***^*** Trimester*** (< 13 weeks)	5 (1.7%)	***2***^***nd***^*** Trimester*** (13–26 weeks)	20 (6.7%)	***3***^***rd***^*** Trimester*** (> 26 weeks)	273 (91.6%)
Maternal fever			123 (42%)		
Asymptomatic			103 (39.5%)		
Severe maternal COVID-19			12 (6.9%)		
Maternal ECMO			3 (1%)		
Maternal total duration of symptoms, days			5.5 (3–10)		
**Neonatal characteristics, N (%), median (IQR)**					
***Mode of delivery***					
Vaginal birth		170 (57%)	Cesarean section		128 (43%)
Multiple gestation			13 (6%)		
Gestational age, weeks			38 (37–39)		
Birth weight, grams			3005 (2660–3440)		
Male		167 (56%)	Female		131 (44%)
**Neonatal ICU admission (*N* = 76)**					
Prematurity		55 (18.4%)	Neonatal hyperbilirubinemia		6 (2%)
Transient tachypnea of newborn		6 (2%)	Congenital heart disease		2 (0.7%)
Hypoxic-ischemic encephalopathy		3 (1%)	Myelomeningocele		1 (0.3%)
Aqueduct stenosis		1 (0.3%)	Epidermolysis bullosa		1 (0.3%)
Early-onset Klebsiella sepsis		1 (0.3%)			
SARS-CoV-2 infection, PCR			2 (0.7%)		
**Type of feeding in the first 6 months of life**					
***Bottle feeding (formula)***	50 (50.7%)	***Breastfeeding (breast milk)***	75 (25.3%)	***Mixed feeding***	71 (24%)
Further hospitalization in the first 8–10 months of life			28 (9.6%)		

Values are expressed as numbers (N) and percentages or medians and interquartile ranges (IQRs). Severe COVID-19 was defined as clinical signs of pneumonia plus SpO_2_ less than 90% in room air or admission to an intensive care unit (ICU) for respiratory support (i.e., high-flow nasal cannula, noninvasive mechanical ventilation, and intubation)

*ECMO* Extracorporeal membrane oxygenation

Among the infants whose parents completed the ASQ-3, 5 (1.7%) were born to mothers diagnosed with SARS-CoV-2 during the first trimester, 20 (6.7%) were born to mothers diagnosed during the second trimester, and 273 (91.6%) were born to mothers diagnosed during the third trimester. Fourteen (4.7%) neonates were born at less than 31 weeks gestation. Twenty-eight infants needed hospitalization beyond the first 8–10 months of life.

**Fig. 2:**
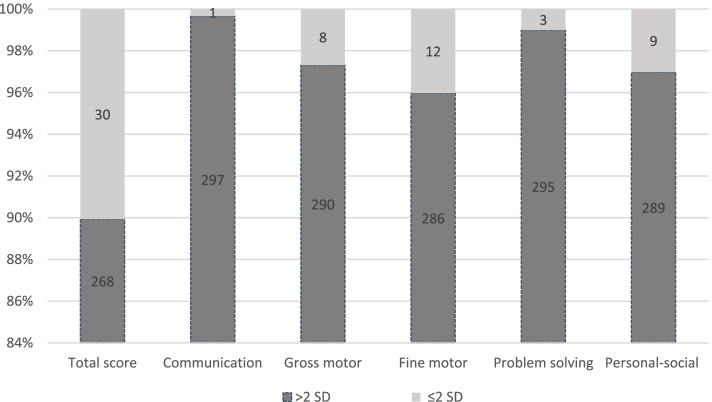
Developmental outcomes using the Ages and Stages Questionnaire, 3rd edition, at 10–12 months of age. A bar graph presenting the total score and subscale scores of the ASQ-3 domains. A score with a standard deviation (SD) ≤ 2 below the population mean implies developmental delays, and a score with an SD > 2 above the population mean implies no delays

Parental questionnaires were collected at a median corrected age of 10.3 months (IQR 10 to 12.7). Developmental delays were identified in 30 (10.1%) of the infants. Of those with developmental delays, 3.3% (1/30) had delays in the communication domains, 27% (8/30) in the gross motor domain, 40% (12/30) in the fine motor domain, 10% (3/30) in the problem-solving domain, and 30% (9/30) in the personal-social domain (Fig. 2).

In the infants with developmental delays, 4 had delays in two or more domains, and none had delays in all domains.

Infants with developmental delays did not differ in terms of maternal age, maternal comorbidities, parental education level, severe maternal COVID-19, or the mode of delivery compared to those with no delays (Table 2). However, the occurrence of developmental delays differed significantly on the basis of the trimester of SARS-CoV-2 infection. Developmental delays were more common in infants born to mothers with COVID-19 infections during the first and second trimesters than in infants born to mothers with COVID-19 infections during the third trimester (*P* < 0.001). Among the infants with developmental delays, 4 (13.3%) were born to mothers with SARS-CoV-2 infections during the first trimester, 6 (20%) were born to mothers with SARS-CoV-2 infections during the second trimester, and 20 (66.7%) were born to mothers with SARS-CoV-2 infections in the third trimester. One (0.4%) of the infants without delays was born to a mother with a SARS-CoV-2 infection in the first trimester, 14 (5.2%) were born to mothers with SARS-CoV-2 infections in the second trimester, and 253 (94.4%) were born to mothers with SARS-CoV-2 infections in the third trimester. Moreover, infants born at less than 31 weeks gestation were more likely to have developmental delays than infants born at more than 31 weeks gestation (13% versus 3.7%, respectively; *P* = 0.002). The neonatal characteristics were similar between the groups. During the study period, only 2 (0.7%) neonates tested positive for SARS-CoV-2 infection, and both had normal developmental outcomes.

**Table 2:** Maternal and neonatal demographic and clinical characteristics by the ASQ-3 results

Variable		Developmental delays *N* = 30	No delays *N* = 268	*P* value
Age, years		33 (30–37)	31 (27–35)	0.089
Parity		2 (2–4)	3 (2–4)	0.373
Gestational diabetes		4 (13.3%)	47 (17.5%)	0.562
Pregnancy-induced hypertension		4 (13.3%)	22 (8.2%)	0.346
Maternal nationality	Kuwaiti	9 (30%)	103 (38.4%)	0.366
	Non-Kuwaiti	21 (70%)	165 (61.6%)	
Educational level	Diploma and above	20 (66.7%)	180 (67.2%)	0.956
Gestational age at SARS-CoV-2 infection	1^st^ trimester	4 (13.3%)	1 (0.4%)	< 0.001*
	2^nd^ trimester	6 (20%)	14 (5.2%)	
	3^rd^ trimester	20 (66.7%)	253 (94.4%)	
Maternal fever		10 (35.7%)	113 (42.8%)	0.47
Asymptomatic		11 (37.9%)	92 (39.7%)	0.858
Severe maternal COVID-19		1 (6.7%)	11 (6.9%)	0.966
Maternal ECMO		0	3 (1.1%)	0.879
Maternal total duration of symptoms		5 (3–10)	7 (3–10)	0.651
Mode of delivery	Vaginal birth	14 (46.7%)	156 (58.2%)	0.226
	Cesarean section	16 (53.3%)	112 (41.8%)	
Multiple gestation		0	13 (6.4%)	0.312
Gestational age, weeks, median (IQR)		38 (37–39)	38 (37–39)	0.922
Gestational age	≤ 31 weeks	4 (13%)	10 (3.7%)	0.002*
	> 31 weeks	27 (90%)	258 (96.2%)	
Birth weight, grams		2950 (2400–3400)	3030 (2680–3440)	0.355
Male		13 (43.3%)	154 (57.5%)	0.139
SARS-CoV-2 infection, PCR		0	2 (0.7%)	0.363
Neonatal diagnosis	Prematurity	6 (20%)	49 (18.3%)	0.818
	Neonatal hyperbilirubinemia	1 (3.3%)	5 (1.9%)	
	Transient tachypnoea of the newborn	2 (6.6%)	4 (1.5%)	
	Hypoxic-ischemic encephalopathy	1 (3.3%)	1 (0.4%)	
	Congenital heart disease	0	1 (0.4%)	
	Myelomeningocele	0	1 (0.4%)	
	Aqueduct stenosis	0	1 (0.4%)	
	Early-onset *Klebsiella* sepsis	0	1 (0.4%)	
	Epidermolysis bullosa	0	1 (0.4%)	
Type of feeding in the first 6 months of life	Bottle feeding (formula)	13 (43.3%)	137 (51.5%)	0.690
	Breastfeeding (breast milk)	9 (30%)	66 (24.8%)	
	Mixed feeding	8 (26.7%)	63 (23.7%)	
Further hospitalization in the 1^st^ 8–10 months of life		2 (6.7%)	26 (9.9%)	0.57

Values are expressed as numbers (N) and percentages or medians and interquartile ranges (IQRs)

Severe COVID-19 was defined as clinical signs of pneumonia plus SpO_2_ less than 90% in room air or admission to the intensive care unit (ICU) for respiratory support (i.e., high-flow nasal cannula, noninvasive mechanical ventilation, and intubation)

*ECMO* Extracorporeal membrane oxygenation

In multivariate logistic regression analysis, adjusting for maternal age, parental educational level, gestational age, birth weight, and the type of feeding in the first six months of life, the risk of developmental delays was higher with first-trimester maternal SARS-CoV-2 infections (aOR: 8.2, 95% CI: 1.1–55.9; *P* = 0.039) and second-trimester maternal SARS-CoV-2 infections (aOR: 8.1, 95% CI: 2.4–27.7; *P* = 0.001) than with third-trimester maternal SARS-CoV-2 infections. Moreover, infants born at less than 31 weeks’ gestation were more likely to have developmental delays (aOR: 7.7, 95% CI: 1.4–45.8; *P* = 0.032) (Table 3).

**Table 3:** Multiple regression analysis of the association of demographic and clinical characteristics with developmental delays (ASQ-3 scores less than 2 standard deviations below the population mean)

Variable		Adjusted odds ratio	95% CI	*P* value
Trimester at SARS-CoV-2 infection	3^rd^	Reference		
	2^nd^	8.1	2.4–27.7	0.001*
	1^st^	8.2	1.1–55.9	0.039*
Maternal age		1.04	0.96–1.1	0.305
Gestational age at birth	≤ 31 weeks	7.7	1.4.-45.8	0.032*
	> 31	Reference		
Maternal education (diploma and above)		1.8	0.59–5.4	0.300
Paternal education (diploma and above)		0.5	0.2–1.3	0.172
Male		1.6	0.68–3.5	0.297
Type of feeding in the first 6 months	Mixed feeding	Reference		
	Bottle feeding (formula)	1.3	0.46–3.4	0.652
	Breastfeeding (breast milk)	0.5	0.14–1.6	0.239

Values are expressed as adjusted odds ratios and 95% confidence intervals (95% CIs)

## Discussions

The fetal inflammatory response (FIRS) due to a maternal SARS-CoV-2 infection can contribute to severe neonatal morbidity, which includes stillbirth, neonatal death, preterm birth, low birth weight, fetal distress, and neonatal asphyxia [19–21]. This was a prospective study reporting neurological assessments of neonates born to mothers with SARS-CoV-2-positive RT–PCR test results during pregnancy using the ASQ-3. The findings of this study indicate that infants born to mothers who test positive for SARS-CoV-2 infection during pregnancy need to be assessed for neurodevelopmental delays.

To assess the developmental attainment of infants born to mothers with SARS-CoV-2-positive test results during pregnancy, this study employed the ASQ-3, a screening tool used for assessing the developmental attainment of infants and young children [18]. This screening tool is used widely in assessing development in children aged 1–66 months at a low cost, with cutoff scores identifying developmental delays. In the current study, an Arabic version of the ASQ-3 was used for parents who were well versed in Arabic. The study cohort included diverse as well as immigrant parents; questionnaires were sent to the parents, and fortunately, the majority of them consented to respond.

In this study, 90% of the neonates at the end of 10–12 months showed normal developmental attainment, and only 10% (*n* = 30) showed developmental delays. The PregCOV-19 living systematic review [1] found that approximately 95% of the neonates born to SARS-CoV-2-positive mothers were reported to be born in good condition. However, it is not clear whether this systematic review included evaluations of neurodevelopmental delays in the neonates. A study from the UK, in which information about educational and behavioral problems was collected from 177 children, found that 25% of the children required support from a nonteaching assistant, 4% required a statement of special educational needs, and 3% were in special schools [22]. Many investigators have studied the effects of various viral infections, excluding SARS-CoV-2, on pregnancy and fetal/neonatal outcomes and have reported that maternal influenza, hepatitis C, varicella-zoster, and other viruses produce distinct fetal brain structures and anatomical clinical pathologies of varying severities [23–26].

In our study, the prevalence of infants with developmental delays was 10%. Compared with a previous study conducted in a similar geographical and cultural setting to Kuwait that used the same ASQ-3 screening tool with healthy children, the prevalence of developmental delays was 15% [27]. The difference could be explained by our smaller collected sample size or could indeed be because the risk of developmental delays among infants born to SARS-CoV-2-infected mothers is not different than that in the general population. Compared with a historical control of infants born before the onset of the COVID-19 pandemic, a prospective study of 255 neonates at 6 months of age using the ASQ-3 reported that exposure to maternal SARS-CoV-2 infection was not associated with lower ASQ-3 scores [28]. A recent study of 199 infants aged less than 10 months in Italy showed that in utero exposure to SARS-CoV-2 infection was not associated with adverse effects on growth or neurological and audiological outcomes. However, 15% of the infants had abnormal ophthalmological evaluations [29]. In both studies, neurological outcomes were assessed in early infancy, an approach that could miss later neurodevelopmental delays. In our study, we evaluated the infants’ neurobehavioral development 10–12 months post-discharge, which is perhaps the adequate duration for effects to appear.

The study data revealed that the majority (91.6%) of the pregnant women had a SARS-CoV-2 infection during their third trimester, whereas 6.7% had a SARS-CoV-2 infection in their second trimester. This finding is in agreement with those of other studies also reporting that most SARS-CoV-2 infections in pregnant women occur in their third trimester [30, 31]. In the current study, only a relatively small number of women were infected during the first trimester. However, first-trimester infections are important as the principal stages of brain development, such as primary neurulation (weeks 3–4), prosencephalic development (months 2–3), and neuronal proliferation (months 3–4), occur during the early stages of pregnancy [32]. In fact, infections with some common pathogens, such as cytomegalovirus (CMV), Zika virus, Rubella virus, *Mycobacterium tuberculosis* (TB), and *Toxoplasma gondii*, during the first and early second trimesters increase the risk of symptomatic infants, with up to 32% having neurological manifestations [33]. Owing to the relatively recent emergence of COVID-19, information related to pregnancy outcomes among women with SARS-CoV-2 infections and the consequences of infant exposure to the virus is very scarce. As more women infected during their first and second trimesters progress in their pregnancy and as their newborns develop, a better understanding of the neurological effects of this novel virus will emerge. In brief, various types of evidence support the theory that maternal infection and/or inflammation occurring during critical periods of fetal development could alter brain structure and function in a time-sensitive manner.

Nevertheless, SARS-CoV-2 infection increases the chances of fetal distress, leading to high incidences of admission to the neonatal intensive care unit (NICU) [31]. Similar to other reported studies, in the current study, 76 out of the 298 (25.5%) neonates born to mothers with SARS-CoV-2 infections during pregnancy required NICU admission [34–37]. However, a systematic review reported that approximately 95% of the neonates born to mothers with SARS-CoV-2 infections during pregnancy were born in good condition [1].

Recent evidence suggests that vertical transmission of SARS-CoV-2, either antenatally or intrapartum, can occur, but it is uncommon. In this study, only 2 (0.7%) neonates tested positive for SARS-CoV-2 infection and did not seemingly experience perinatal complications. The majority of the neonates (296 out 298) were negative for SARS-CoV-2 infection. Our observation is in agreement with those of other studies [30, 38, 39], which implies that the consequences of SARS-CoV-2 infection on neurodevelopment were largely due to in utero effects rather than direct effects on the fetus. The reason for the low vertical transmission rate is that the placenta has low expression of the canonical receptors that are necessary for virus entry, which may explain the rarity of the vertical transmission of SARS-CoV-2 [40]. Importantly, teratogenic effects of SARS-CoV-2 on the fetuses were not observed, which is in contrast to other viral infections, such as Middle East respiratory syndrome coronavirus, CMV, herpes virus, Zika virus, and Rubella virus infections [40–44].

Of note, the role of cytokine storms, particularly the role of IL-6 in the pathogenesis of neurodevelopmental disorders, is not fully elucidated; however, in a longitudinal study, alterations in brain architecture, executive function, and working memory abilities were reported in 2-year-old children exposed to increased IL-6 levels during pregnancy [45]. An analysis of 214 patients with SARS-CoV-2 infections revealed that 36% had neurological symptoms [46]. Many case–control studies have demonstrated that elevated maternal cytokine levels affect the fetal brain, causing neurological disease [44–47]. The FIRS, due to increased IL-6 levels following a maternal SARS-CoV-2 infection [13], may induce a wide range of adverse neurodevelopmental sequelae, such as autism, psychosis, and neurosensory deficits, later in life [48], similar to certain bacterial infections [49]. However, long-term longitudinal studies are required to validate these associations.

Moreover, the risk of neurological effects on neonates differs by the trimester in which SARS-CoV-2 infection occurs [50]. In this study, developmental delays were relatively more prevalent when infection occurred in the first and second trimesters than when infection occurred in the third trimester (*P* < 0.001). The neonatal characteristics were similar between the groups. Moreover, infants born at less than 31 weeks gestation were more likely to have developmental delays than those born at more than 31 weeks gestation (13% versus 3.7%, respectively; *P* = 0.002). However, this finding in our series needs to be considered with caution, as preterm infants are more likely to have adverse neurodevelopmental outcomes than full-term infants [51].

### Strengths and limitations

This is perhaps the first study to report neurological assessments, using the ASQ-3, of neonates born to mothers with SARS-CoV-2-positive RT–PCR test results during pregnancy. The participating mothers were from the local area, which enabled good tracking for follow-ups. The participants were educated and responded to the ASQ-3 appropriately. Thus, these points may be regarded as strengths of the study. The findings of the study may be considered reliable, as the cohort of parents who participated in the study was well educated, and women were young (median age: 31 years). Three-quarters of the women did not have pregnancy-induced hypertension or gestational diabetes.

This study also has a few limitations, the most important aspects of which are related to the sample size and methodology. The most important weakness of our study was that we did not have a control group of mothers without COVID-19. For stronger conclusions, a larger and statistically valid sample size is needed. Nevertheless, this initial study may help healthcare authorities understand the need to assess the developmental attainment of infants born to mothers with SARS-CoV-2-positive test results during pregnancy. The ASQ-3, although a good screening tool, has limitations, and its results may not be strongly comparable to those of other tools, such as the Bayley III test; therefore, further larger studies with formal assessments of neurodevelopment are warranted.

## Conclusions

This study was a hypothesis-generating study, and the overall findings based on a screening tool seemed favorable. Some concerns were raised regarding neurodevelopmental effects on fetuses born to mothers with SARS-CoV-2-positive test results during pregnancy in 10% of the infants. A majority of the pregnant women had SARS-CoV-2 infections during their third trimester. Developmental delays were more prevalent when SARS-CoV-2 infection occurred in the first and second trimesters than when it occurred in the third trimester. Infants born at less than 31 weeks gestation were more likely to have developmental delays than those born at more than 31 weeks gestation. However, since we did not have a control group, firm conclusions on the findings of our study cannot be provided.

Further larger studies with formal developmental assessments are warranted to study the effect of SARS-CoV-2 on the developing brain.  

## Data Availability

The analysis code compiled using Stata software is available from the corresponding author. Data sharing with readers is not available because of the requirements of the Ethical Committee of the Ministry of Health of Kuwait.

## Abbreviations

MIS-N: Multisystem inflammatory syndrome in neonates
SARS-CoV-2: Severe acute respiratory syndrome coronavirus-2
COVID-19: Coronavirus disease 2019
RT–PCR: Reverse transcriptase-polymerase chain reaction
CDC: Centers for Disease Control
CRP: C-reactive protein
IL: Interleukin
FIRS: Fetal inflammatory response
